# TCGAnalyzeR: An Online Pan-Cancer Tool for Integrative Visualization of Molecular and Clinical Data of Cancer Patients for Cohort and Associated Gene Discovery

**DOI:** 10.3390/cancers16020345

**Published:** 2024-01-13

**Authors:** Talip Zengin, Başak Abak Masud, Tuğba Önal-Süzek

**Affiliations:** 1 Department of Molecular Biology and Genetics, Mugla Sitki Kocman University, Mugla 48000, Türkiye; talipzengin@mu.edu.tr; 2 Department of Bioinformatics, Mugla Sitki Kocman University, Mugla 48000, Türkiye; basakkabakk@gmail.com

**Keywords:** clinical data integration, cancer subcohort analysis, TCGA data visualization, driver mutations prediction, copy number variations in cancer, transcriptome analysis, oncology research platforms

## Abstract

**Simple Summary:**

TCGAnalyzeR provides a novel web site integrating 123 pre-computed pan-cancer cohorts (i.e., microsatellite instability, immune, metastasis, PAM50, Triple Negative breast cancer, idh1-mutated glioblastoma, etc.), along with our own iCluster+ subcohorts, computed based on pre-processed single-nucleotide variations, copy number variations, differential expression, miRNA, methylation, and clinical data. TCGAnalyzeR interface provides an optimized and fully customizable experience to each user, enabling the selection of their own “My patients” or “My genes” to a clipboard. Several use cases of the web site are presented as Help documents.

**Abstract:**

For humans, the parallel processing capability of visual recognition allows for faster comprehension of complex scenes and patterns. This is essential, especially for clinicians interpreting big data for whom the visualization tools play an even more vital role in transforming raw big data into clinical decision making by managing the inherent complexity and monitoring patterns interactively in real time. The Cancer Genome Atlas (TCGA) database’s size and data variety challenge the effective utilization of this valuable resource by clinicians and biologists. We re-analyzed the five molecular data types, i.e., mutation, transcriptome profile, copy number variation, miRNA, and methylation data, of ~11,000 cancer patients with all 33 cancer types and integrated the existing TCGA patient cohorts from the literature into a free and efficient web application: TCGAnalyzeR. TCGAnalyzeR provides an integrative visualization of pre-analyzed TCGA data with several novel modules: (i) simple nucleotide variations with driver prediction; (ii) recurrent copy number alterations; (iii) differential expression in tumor versus normal, with pathway and the survival analysis; (iv) TCGA clinical data including metastasis and survival analysis; (v) external subcohorts from the literature, curatedTCGAData, and BiocOncoTK R packages; (vi) internal patient clusters determined using an iClusterPlus R package or signature-based expression analysis of five molecular data types. TCGAnalyzeR integrated the multi-omics, pan-cancer TCGA with ~120 subcohorts from the literature along with clipboard panels, thus allowing users to create their own subcohorts, compare against existing external subcohorts (MSI, Immune, PAM50, Triple Negative, *IDH1*, miRNA, metastasis, etc.) along with our internal patient clusters, and visualize cohort-centric or gene-centric results interactively using TCGAnalyzeR.

## 1. Introduction

With the decreasing cost and increased availability of the new generation of sequencing techniques and their power to simultaneously detect more than one gene variant in the clinic, many genetic tests have been released to the market and approved for clinical diagnosis by the FDA. Examples of tests in clinical use are the Oncomine Dx Target Test [1], which tests the sequence variations of 46 genes on DNA and RNA for lung cancer, the Oncotype DX test [2], which tests the 21 mutations for breast cancer, and the PAM50 test [3], which tests the expression data of 50 genes in breast cancer. Yet, due to several recent clinical studies showing that the powers of these tests are not comparable to conventional single gene tests, there is an open question on how to analytically compare the clinical performance of personalized oncology tests.

The sheer scale and complexity of The Cancer Genome Atlas (TCGA) data [4] offers great potential for scientific discovery, but the challenges to effective use of this valuable resource by biologists and clinicians have led to the development of several visualization tools such as cBioPortal [5,6], Firebrowse [7], and University of California, Santa Cruz (UCSC) Xena [8]. These visualization tools do not serve as data repositories but rather aim to create an integrated visualization of TCGA. Among these tools, cBioPortal is the most preferred due to its interactive exploration of larger and up-to-date cancer datasets. OncoKB [9] is another precision oncology knowledge base that allows searching and comparing of drug response data from different TCGA cohorts, yet the visualizations are limited by variant effects. Although the ICGC web portal [10] allows patient/gene subsetting of TCGA cohorts and provides survival and set operation visualization of cohorts, it does not allow comparison of cohorts compiled from the literature generated by other research groups. Only the Coral web application [11], similar to TCGAnalyzeR, incorporates a few (MSI, tumor-purity and immune) subcohorts from the literature, yet it does not allow for comparison against each other and it does not allow for their projection onto Oncoplots. Most of these comparable tools provide access to raw data only, with limited additional pre-processing. TCGAnalyzeR, in contrast, enables users to project any cohort out of an extensive set of 123 pre-loaded patient subcohorts onto Oncoplots/Oncogrids of patient mutations, in addition to survival and subsetting options. Oncoplot/Oncogrid visualizations are especially critical for oncologists who use FDA-approved diagnostic gene panels, such as the Oncotype DX test or PAM50, and want to validate these tests’ predictive power against a wide spectrum of existing TCGA cohorts. The comparison of the existing TCGA visualization web tools against TCGAnalyzeR is summarized in Table 1. 

To address this limitation, we developed an interactive R Shiny web application for the analysis and visualization of four data categories across 33 cancer types. Users can visualize the results of preprocessed analysis of Simple Nucleotide Variations (SNVs), Copy Number Variations (CNVs), differential gene expression in tumor versus normal samples, and clinical data of TCGA projects from the National Cancer Institute’s (NCI) Genomic Data Commons (GDC) [12]. Moreover, users can compare patient clusters determined using an iClusterPlus R package [13] with expression-based survival risk groups [14,15] and curated subtypes, such as immune subtypes [16], Triple Negative Breast Cancer (TNBC) subtypes [17], PAM50 subtypes [18], Microsatellite Instability (MSI)-related subgroups and several data type clusters from BiocOncoTK [19,20], and curatedTCGAData (version 1.20.1) R packages [21]. While gathering many of these subcohorts in a clinical setting can present challenges, such as metastasis patients with primary tumor data or MSI-H in non-endometrial cancers like BRCA, the vast size of TCGA enables the analysis of these rare subcohorts. Furthermore, users can create custom subcohorts based on genomic analyses and/or clinical data, including metastasis organs/tissues to subset data visualization. Users can also create gene sets for data type and/or pan-cancer comparisons. For each cancer, whenever available, sample types, survival risk groups (Low-risk/High-risk), and pre-computed or curated patient clusters can be used for filtering patients. The main novelty of our tool is its ability to integrate many published subcohorts at a single pan-cancer interface, allowing the users to generate their own custom patient sub-cohorts and/or gene sets using interactive graphical representations via clipboard functionality.

## 2. Materials and Methods

### 2.1. TCGA Data

Publicly available hg38 data, including SNV, CNV, Transcriptome Profiling, microRNA, Methylation, and clinical data of 33 cancer types from The Cancer Genome Atlas (TCGA) projects, were downloaded on 6 March 2022 from NCI GDC [12] using TCGAbiolinks R package [22].

### 2.2. Pre-Computed Molecular Data Analysis

#### 2.2.1. SNV Analysis

Potential driver mutated genes, with their roles as a tumor suppressor or oncogene, were determined by SomInaClust R package [23] using a mutation annotation format (maf) file generated by mutect2 pipeline. With the “Somatic Driver Mutations” option, the user can see the significant mutated genes ranked by their *q*-value. This option is only available for the “SNV Analysis” category. Statistical methods implemented for SNV analysis are described in more detail in our previous publications [14,15] and the R codes are provided in the GitHub repository.

#### 2.2.2. CNV Analysis

Significant recurrent copy number variations were identified by GAIA R package [24]. NCBI IDs and Hugo Symbols of the genes on chromosomal regions with altered copy numbers were determined using GenomicRanges [25] and biomaRt [26] R packages. Statistical methods implemented for CNV analysis are described in more detail in our previous publications [14,15], and the R codes are provided in the GitHub repository.

#### 2.2.3. Gene Expression Analysis

Two different analyses were performed using paired tumor samples against tumor-adjacent normal samples of patients with both sample types (Paired), or tumor samples of all patients against normal samples of patients who have both sample types (All), if it was available for a particular cancer. For cancers with paired tumor samples, differentially expressed genes were determined using normalized HTseq counts, by limma-voom method with a ‘duplicate correlation’ function from edgeR [27] and limma [28] R packages. Ensemble IDs were converted to NCBI IDs and Hugo Symbols using the biomaRt package [26]. For 11 cancers out of 33 cancer types, there is no patient sample with tumor adjacent normal tissue that exists, therefore we created the (All) option for all cancers using the normalized HTseq counts by the TMM method, followed by a Log2 transformation. Genes with consistently zero or low counts were filtered out. Statistical methods implemented for paired differential expression analysis are described in more detail in our previous publication [14,15] and the R codes are provided in the GitHub repository.

#### 2.2.4. Pathway Enrichment

Pathway enrichment and visualization was performed for each analysis using a clusterProfiler R package [29]. Statistical methods implemented for CNV analysis are described in more detail in our previous publication [14,15], and the R codes are provided in the GitHub repository.

#### 2.2.5. Pre-Computed Patient Clusters and Curated Subcohorts from the Literature

Although many pan-cancer subcohorts based on TCGA data, such as Microsatellite Instability (MSI) clusters [19,20] and immune clusters [16], have been published in high-impact journals, only few tools (i.e., Coral) have integrated them into their visualizations. The Coral web application [11] integrated only a few of the literature-curated cohorts, such as MSI, tumor-purity and immune subcohorts, yet it does not allow comparison of these subcohorts against each other and it does not allow projection of these subcohorts onto Oncoplots/Oncogrid visualizations. As of January 2024, there was no web tool in the literature that allowed for visual comparison of a large number of patient subcohorts with each other. To address this need, TCGAnalyzerR integrated 123 external patient cohorts from the literature into the web interface, enabling efficient filtering and facilitating cross-comparative analysis of multiple subcohorts in parallel. TCGAnalyzeR provides an interactive visual analysis of several patient cohorts. (i) Survival Risk Groups: we provide low-risk or high-risk patient groups determined by expression-based gene signature analysis for Lung Adenocarcinoma (LUAD), Lung Squamous Cell Carcinoma (LUSC), and Colon Adenocarcinoma (COAD) [14,15]. (ii) iClusters: we clustered patients using their raw SNV, CNV, gene expression, miRNA expression and methylation data of tumor samples which have all 5 types of data using the iClusterBayes method [30]. (iii) Curated subcohorts: Immune, TNBC, MSI, PAM50 subtypes are downloaded from original publications [16,17,18] and for fifteen cancer types, previously published TCGA cohorts of the individual tumor types were retrieved from the curatedTCGAData R package (version 1.20.1) [21]. Patient clusters based on Microsatellite Instability (MSI) were compiled using BiocOncoTK [19,20], and Immune clusters [16] were compiled from its original publication for all 33 cancers. Metastasis site information is brought together in-house from BCR Biotab clinical patient data, BCR Biotab clinical new tumor event data, and BCR Biotab clinical new tumor event follow-up data using the TCGAbiolinks R package.

#### 2.2.6. Survival Analysis

Real-time Kaplan–Meier (KM) survival analysis is conducted using the survival R package [31] and is based on overall survival data for patients of interest with selected clinical features. Data input and tabular reading are facilitated by the readr [32] R package.

#### 2.2.7. Visualization

The TCGAnalyzeR front-end was implemented using javascript-based R packages with an interactive dashboard enabling users to select cancer types, data types, risk groups, and patient cohorts using heatmaply, g3viz, and highcharter R packages [33,34,35]. All visualizations are interactive and customizable by the user through the filtration options with “My genes” and/or “My patients” panels, enabling them to copy genes and/or patients of interest to the clipboard. For BRCA, OncotypeDX gene identifiers are provided to users as an example use case of the “My genes” clipboard. TCGAnalyzeR currently supports the tab separated values (TSV) file type for downloading tables and a high-resolution PNG format for downloading figures. The help page provides links to the original publications of the external subcohorts. An animated image of usage examples has been placed in the help section.

#### 2.2.8. Performance Optimization

For the web performance profiling of all the tools, the profvis package is utilized to inspect the call stack, identify, and optimize the most memory- and (computationally) time-consuming parts of each module. Since its launch on 1 January 2022, although not published, TCGAnalyzeR has been accessed by an average of 79 unique user IPs per day.

## 3. Results

**Figure 1:**
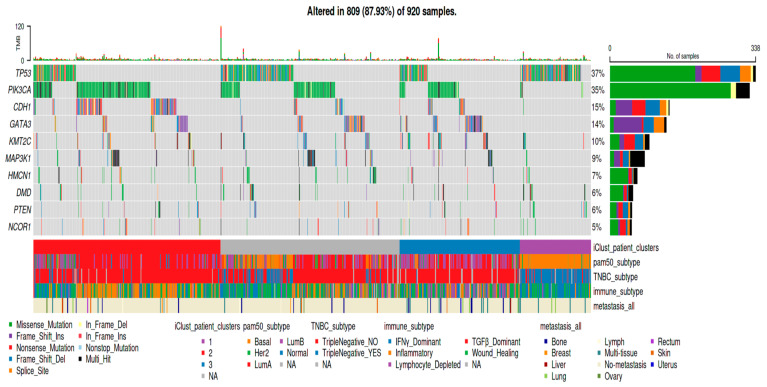
Oncoplot of candidate driver genes with our own pre-computed patient clusters and curated subcohorts from the literature. Top 10 significant candidate driver genes with mutations determined by SomInaClust R package. Bottom annotations show the iClusters, metastasis organs, and subcohorts curated from the literature.

The TCGAnalyzeR web application offers simple nucleotide (SNV) analysis as its first step. We present two data sets for SNV analysis: “Somatic Driver Mutations” predicted by the SomInaClust R package and “All” mutations from the original maf file without any analysis. The Oncoplot in Figure 1 shows candidate driver genes with their percentages in tumor samples of Breast Invasive Carcinoma (BRCA) with annotations regarding patient iClusters, PAM50, TNBC, immune subtypes, and metastasis organs/tissues. iCluster 1 is highly correlated with the Basal and TNBC subtype. Wound-healing and IFNɣ-dominant immune subtypes gather around iCluster 1. iCluster 2 is mostly correlated with the Luminal A subtype and Inflammatory immune subtype. iCluster 3 seems to be a mixture of estrogen receptor positive Luminal A and Luminal B subtypes and heterogenous immune subtypes. Moreover, both iCluster 2 and iCluster 3 are not TNBC subtypes. On the other hand, iCluster 1 shows a highly different mutation pattern than other clusters. iCluster 1, together with basal and triple-negative subtypes, has a higher prevalence of TP53 mutations with very few mutations of *PIK3CA*, *CDH1*, *GATA3*, *KMT2C*, or *MAP3K1* genes. In addition, mutations of *TP53*, *CDH1*, and *GATA3* genes are mutually exclusive. Furthermore, the presence of metastases is heterogeneous across iClusters, PAM50, TNBC subtypes, and immune subtypes (Figure 1), while it is highly correlated with higher tumor stages.

**Figure 2:**
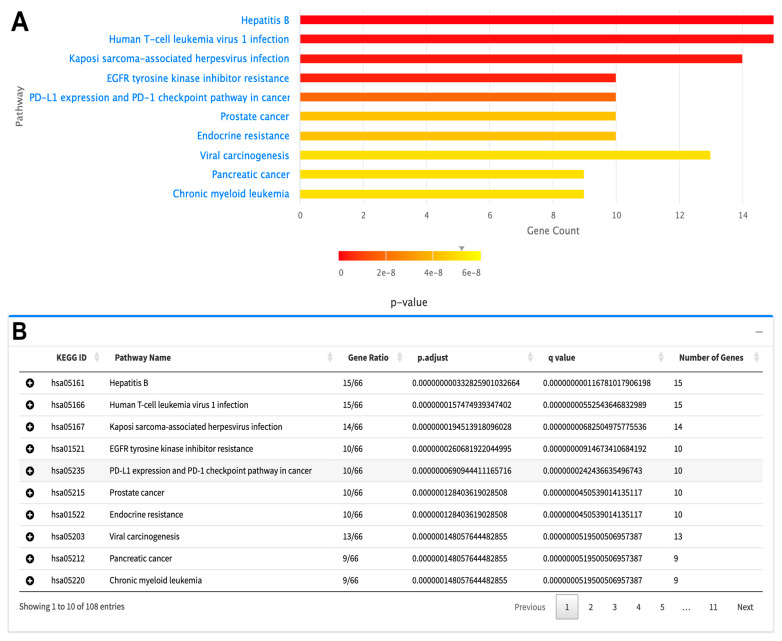
A sample pathway enrichment plot of candidate BRCA driver genes as downloaded from the TCGAnalyzeR website. (**A**) Bar plot showing top 10 significant pathways of BRCA candidate driver genes determined by SomInaClust R package. (**B**) Pathway enrichment table presenting KEGG ID, genes in significant pathways with adjusted *p*-value and *q*-value.

Pathway enrichment of candidate driver mutated genes is shown as a bar graph in Figure 2A. Significant pathways of driver genes are highly cancer-related pathways, such as EGFR tyrosine kinase inhibitor resistance, PD-L1, and PD-1 pathways in cancer, prostate cancer, pancreatic cancer and chronic myeloid leukemia pathways. Pathway enrichment analysis also supplies a table showing KEGG IDs, with related genes and *p*/*q*-values (Figure 2B).

**Figure 3:**
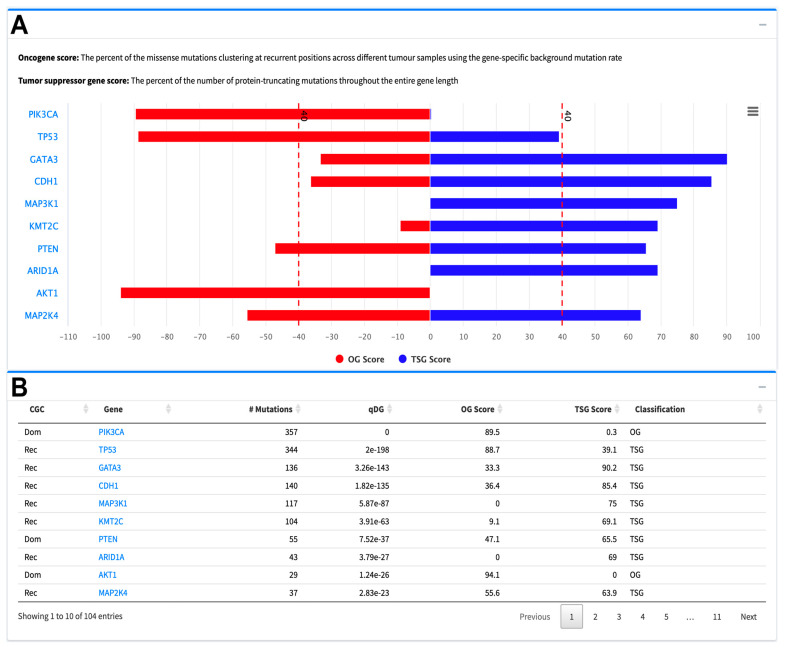
Sample visualization plots of SomInaClust predictions of candidate driver genes as downloaded from the TCGAnalyzeR website. (**A**) Sample web site output showing the pyramid plot of the Oncotype DX genes which were predicted as candidate driver genes with calculated oncogene (OG) and tumor-suppressor (TSG) scores for BRCA by SomInaClust R package. (**B**) Sample web site output showing the SomInaClust analysis results for BRCA with number of mutations, OG score, TSG score, red dashed line representing the SominaClust score threshold of 40 and *q*-value (qDG). CGC: COSMIC cancer gene census, Rec: Recessive (TSG), Dom: Dominant (OG).

The SomInaClust R package determines candidate driver mutated genes with their potential roles as tumor suppressors (TSG) or oncogenes (OG) with predicted scores [23]. The pyramid plot in Figure 3A summarizes the TSG score and OG score of candidate driver genes ranked by their analysis *q*-values. Some genes may have both an OG score and a TSG score over the threshold score of 40, in that case, SomInaClust considers the COSMIC cancer gene census (CGC) information (Figure 3B).

**Figure 4:**
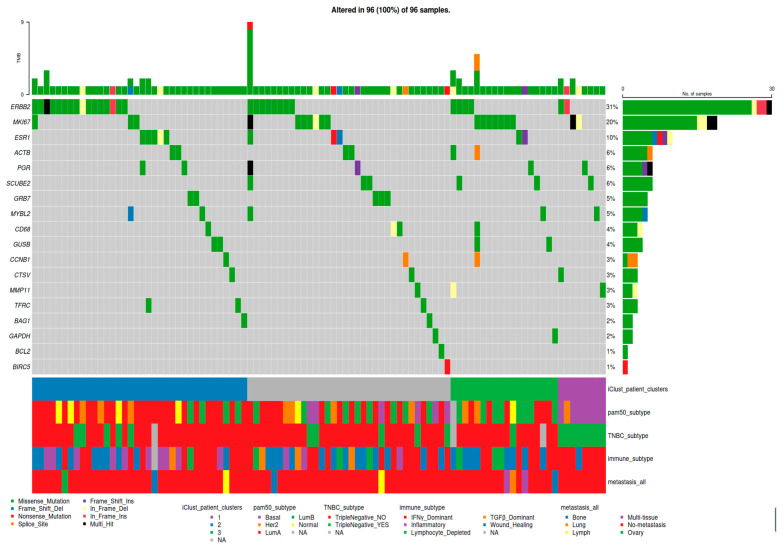
A sample Oncoplot of BRCA Oncotype DX genes with patient clusters and pre-computed subcohorts as downloaded from the TCGAnalyzeR website. Mutations of Oncotype DX genes with annotations showing the patient iClusters and pre-computed BRCA patient subcohorts curated from the literature.

The “My genes” clipboard panel of TCGAnalyzeR allows for the modification of plots in order to show genes of interest. For example, Figure 4 shows the mutation pattern of the Oncotype DX gene set together with clinical annotations. iCluster 2, Luminal A subtype, and Her2 subtypes are highly related with *ERBB2* (*HER2*) mutations. Additionally, iCluster 1 has fewer mutations than the other two iClusters. Moreover, mutations of Oncotype DX genes are mostly mutually exclusive (Figure 4).

**Figure 5:**
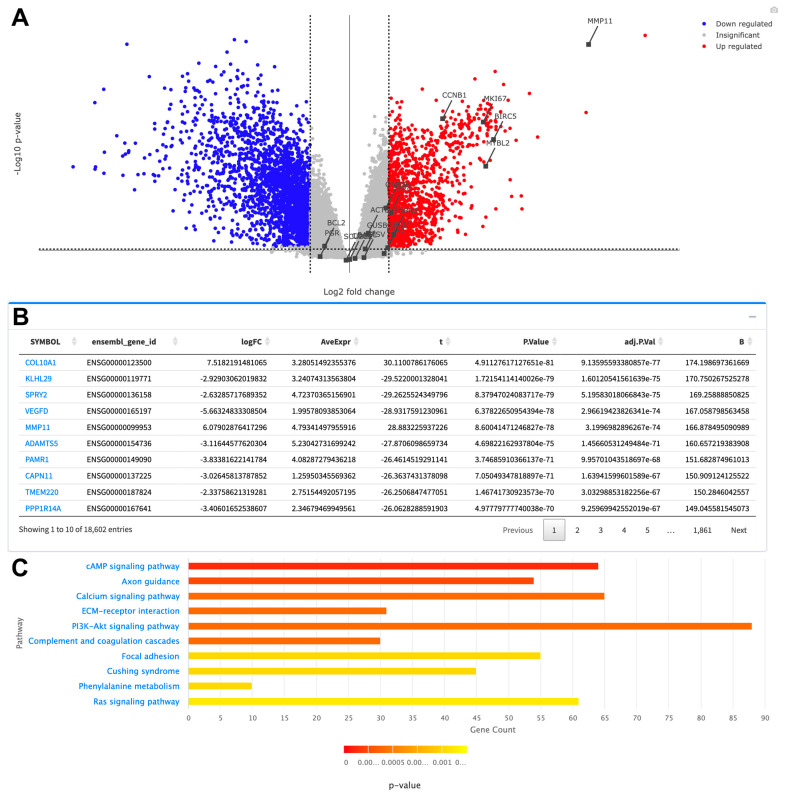
Sample visualizations of differential expression of genes in BRCA tumor samples versus normal samples as downloaded from the TCGAnalyzeR website. (**A**) Volcano plot showing up-regulated and down-regulated genes with -log10 conversion of *p*-values. Oncotype DX genes are highlighted on the graph with gene identifiers connected to the black dots. Black dashed line designates the logFC threshold of 1. Camera image on the upper right corner lets the users download the figure in png format. (**B**) Differential expression results table presenting gene symbols, fold changes (logFC) and adjusted-*p*-values. (**C**) Bar plot showing pathway enrichment of differentially expressed genes.

Transcriptome analysis module provides differential expression analysis (DEA) of RNAseq data by comparing the expression of genes in primary tumor samples against adjacent normal samples. We present two result options for this analysis: “Paired” as a comparison of tumor samples against their own paired normal or “All” as a comparison of tumor samples against a normal sample subset of patients, if such is available for the particular cancer. The volcano plot in Figure 5A summarizes the differential expression analysis of paired BRCA samples and Oncotype DX genes that are highlighted through the “My Genes” panel. Figure 5B presents the table showing the details of DEA with gene symbols, fold change (logFC), and *p* values of significantly differentially expressed genes ranked by *p*-value. Pathway enrichment of differentially expressed genes showed that these genes play a role in focal adhesion and ECM-receptor interaction, which can be related with metastasis, Ras signaling, PI3K-Akt signaling, cAMP signaling, and Phenylalanine metabolism pathways, which are related with cell growth (Figure 5C).

The metastasis-related gene *MMP11* and proliferation-related genes *BIRC5*, *MYBL2*, *MKI67* (Ki67), *AURKA* (*STK15*), *CCNB1*, and *ERBB2* (*HER2*) from the Oncotype DX gene set exhibit significant up-regulation in tumor samples of breast cancer (BRCA). However, hormone-related genes (*BAG1*, *BCL2*, *CD68*, *ESR1* (*ER*), *GSTM1*, *PGR*, *SCUBE2*) do not show significant differential expression among all tumor samples (see Figure 5A).

**Figure 6:**
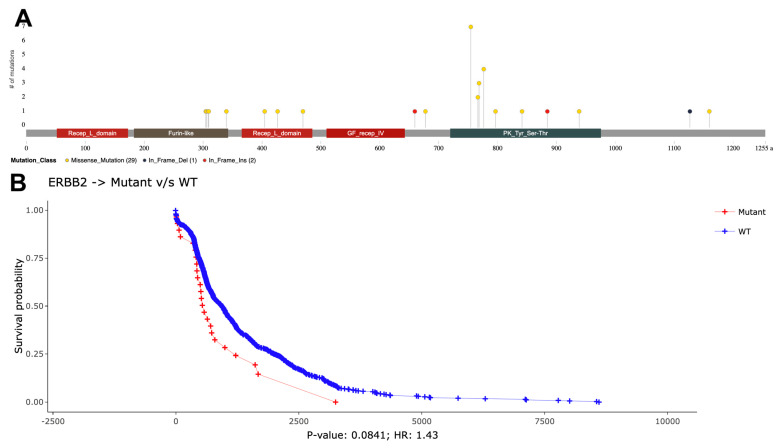
A sample analysis of *ERBB2* (*HER2*) mutation visualizations as downloaded from the TCGAnalyzeR website. (**A**) Lollipop plot showing mutations of *ERBB2* gene among BRCA tumor samples. (**B**) Overall survival analysis of BRCA wild-type versus mutated *ERBB2* in BRCA tumor samples.

Focusing on the *ERBB2* gene, which was predicted as a driver oncogene, the positions of mutations can be visualized using the lollipop plot in Figure 6A. Most of the mutations in the *ERBB2* gene are in the kinase domain (see Figure 6A). These mutations are mostly missense on protein positions 755 (*n* = 7), 767 (*n* = 2), 769 (*n* = 3), 777 (*n* = 4), 797 (*n* = 1), 842 (*n* = 1), and 939 (*n* = 1) and in frame insertion on protein position 885 (*n* = 1). From these mutations, D769H (*n* = 1), D769Y (*n* = 2), V777L (*n* = 4), and V842I (*n* = 1) mutations are activating mutations and L755S (*n* = 5) causes lapatinib resistance [36]. Mutations on the *ERBB2* gene in tumor samples cause lower survival probability with a 1.43 hazard ratio (*p* = 0.08) (Figure 6B). This is related to the finding that the existence of a mutation in the *ERBB2* gene is one of the prognostic indicators of survival for patients with a primary invasive lobular carcinoma subtype of breast cancer [37].

**Figure 7:**
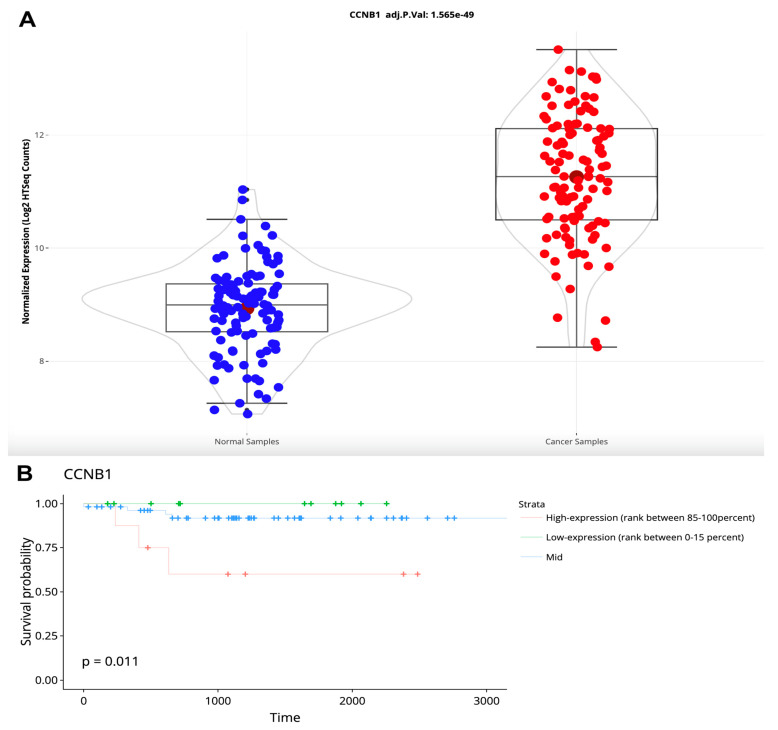
A sample expression analysis of *CCNB1* expression visualizations as downloaded from the TCGAnalyzeR website. (**A**) Violin plot presenting log2 transformed normalized mRNA expression of *CCNB1* in adjacent normal and BRCA tumor samples with adjusted *p*-value. Blue and red circles represent the normalized expression level of each patient’s tumor adjacent normal tissue and tumor tissue respectively (**B**) Overall survival analysis of expression levels of *CCNB1* in BRCA tumor samples. + sign indicates a censored patient. Green, blue and red colors represent the survival day of patients with CCNB1 expression between 0–15%, 15–85% and 85–100% of all sorted normalized CCNB1 expression values respectively.

We checked the expression levels of *CCNB1*, which is one of the upregulated OncotypeDX genes in tumor samples, versus adjacent normal samples (from paired DEA) (Figure 5A). *CCNB1* is expressed in tumor samples at a significantly higher rate than in their adjacent normal samples (*p* = 1.565 × 10^−49^) (Figure 7A). Moreover, patients with higher expression of *CCNB1* have significantly higher survival probability (*p* = 0.011) (Figure 7B), which is correlated with the finding that high CCNB1 protein expression was associated with poor clinical outcomes [38].

**Figure 8:**
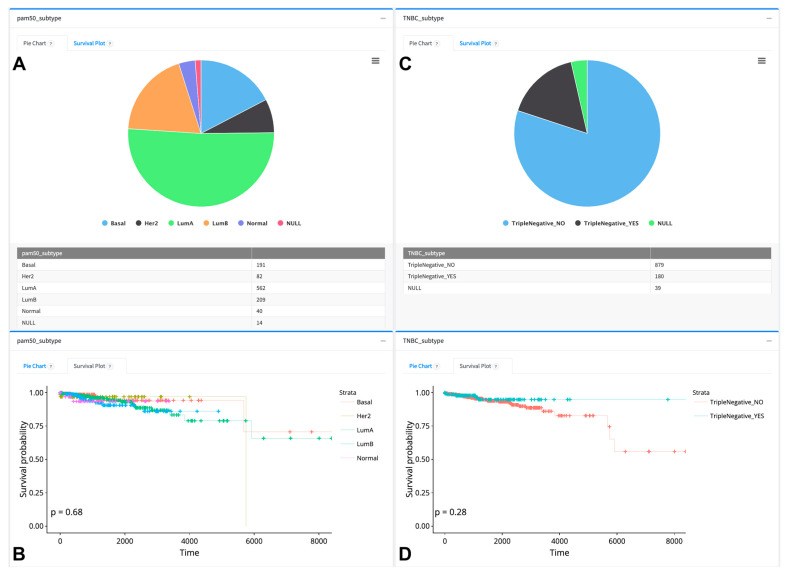
Sample of pie chart and survival analysis visualizations of BRCA PAM50 and TNBC subtypes as downloaded from the TCGAnalyzeR website. (**A**,**C**) Pie chart representation and number of BRCA patients in subtype groups. (**B**,**D**) Survival analysis of subtype groups. Each color dot in the survival analysis (**B**,**D**) represent a patient’s number of survived days for each dynamically selected patient group. + sign indicates a censored patient.

**Figure 9:**
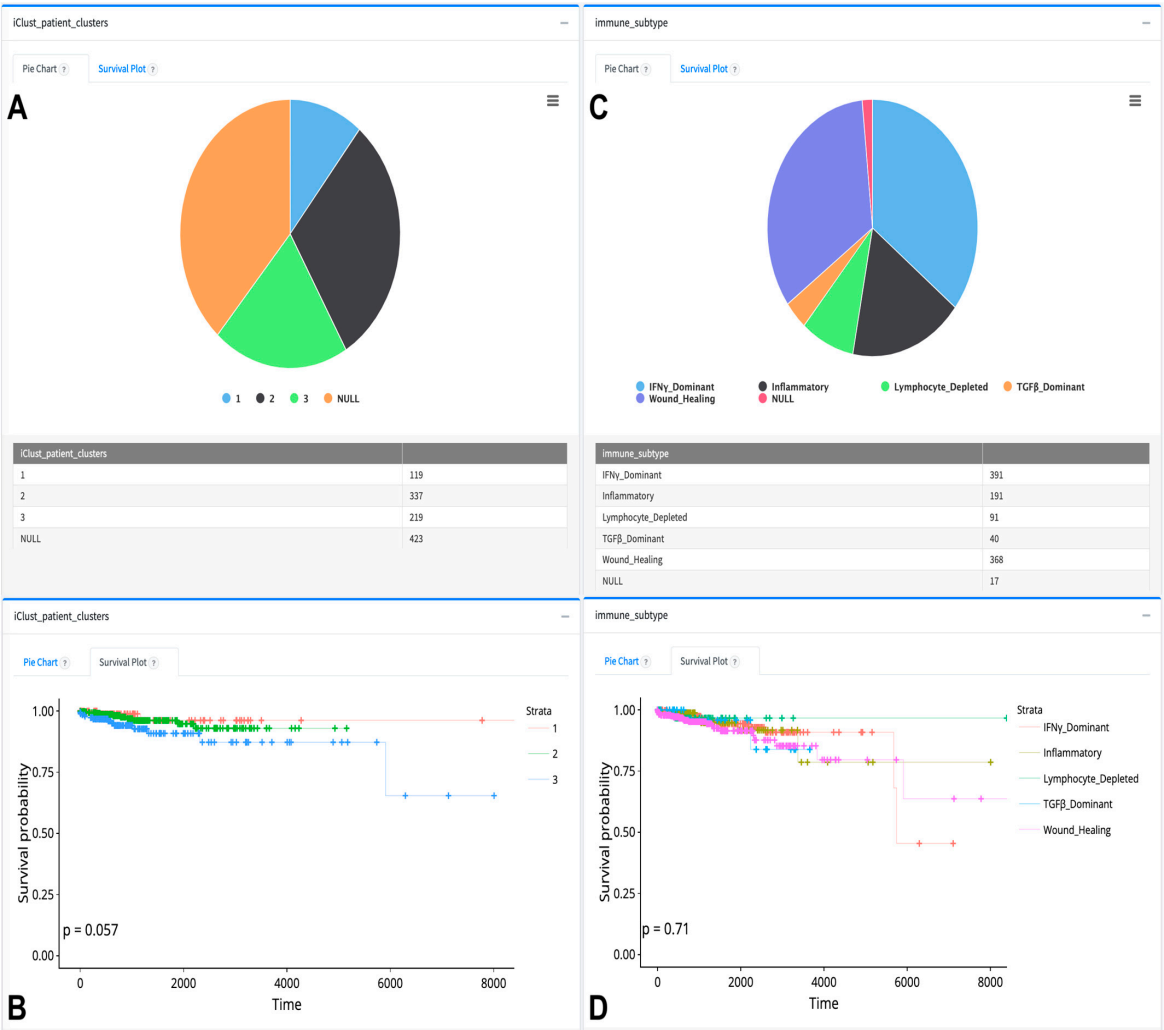
Sample of pie charts and survival analysis of iCluster and Immune subtypes as downloaded from the TCGAnalyzeR website. (**A**,**C**) Pie chart representation of BRCA patients who have metastasis information. (**B**,**D**) Survival analysis of BRCA patient groups with different tissue metastasis. Each color dot in the survival analysis (**B**,**D**) represent a patient’s number of survived days for each dynamically selected patient group. + sign indicates a censored patient.

Clinical data analysis comprises pie chart visualization and survival analysis of clinical features using our patient clusters and pre-computed patient subcohorts gathered from the literature. Figure 8 and Figure 9 depict the visualization of proportions and the survival status of PAM50, TNBC, iClusters, and immune subtypes. iClusters exhibited a differential survival probability close to the significance level (*p* = 0.057); however, PAM50, TNBC, and immune subtypes did not show differential survival probabilities (*p* = 0.68) (see Figure 8 and Figure 9).

**Figure 10:**
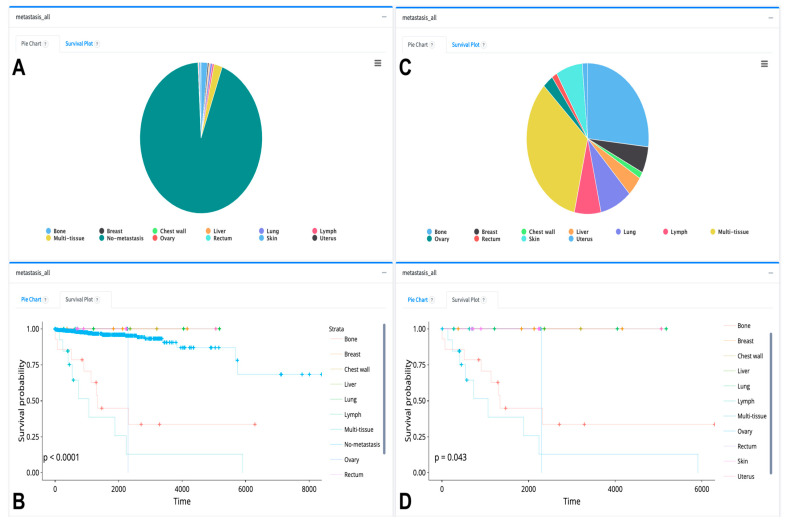
Sample of pie chart and survival analysis visualizations of patients with metastasis as downloaded from the TCGAnalyzeR website. (**A**,**C**) Pie chart representation of BRCA patients who have metastasis information. (**B**,**D**) Survival analysis of BRCA patient groups with different tissue metastasis. Each color dot in the survival analysis (**B**,**D**) represent a patient’s number of survived days for each dynamically selected patient group. + sign indicates a censored patient.

When we parsed the metastasis organs in breast cancer clinical data, 71 patients with primary tumors contain metastasized organ information. Using the TCGAnalyzeR clinical tab, one can filter the pie chart and survival analysis using metastasis conditions by excluding the “No-metastasis” data. The final filtered pie chart shows that most of the 71 breast cancer patients have metastasis to bone or multi-tissue, and these patients have significantly less overall survival probability (Figure 10).

Radial slices of the pie charts are clickable, letting the user add the corresponding patient subsets to the “My Patients” clipboard panel. Furthermore, users can customize a variety of plots such as survival plot, volcano plot, box plot, heatmaps, lollipop plot, and pie charts for the purpose of discovering common molecular profiles for precision oncology. Each plot and data table are downloadable for use in articles.

## 4. Discussion

Several web portals facilitating analysis on TCGA data have been developed and widely used, such as the Genomic Data Commons (GDC) data portal [12], ICGC data portal [10], and CPTAC data portal [39]. The cBioPortal is an open-access, open-source resource for interactive exploration of multidimensional cancer genomics data sets [5,6] providing gene-centered query and visualization functions across multiple cancers. IntOGen is another similar framework for automated comprehensive knowledge extraction based on mutational data from sequenced tumor samples from TCGA patients [40]. However, we provide pre-performed SNV, CNV, and differential expression analyses with large sets of our own patient clusters and pre-computed patient subcohorts. We present signature-based clustering using the Generalized Linear Model for three cancer types (LUAD, LUSC, and COAD). For all 33 cancer types, the immune and MSI-sensor scores of all patients are retrieved from their original publications. For breast cancer (BRCA), PAM50 and TNBC patient cohorts are retrieved from their original publications and metastasis data is retrieved from BCR Biotab. For fifteen cancer types, previously published TCGA cohorts of the individual tumor types are retrieved by a curatedTCGAData R package [21]. iClusterPlus-based patient cohorts are generated for 32 cancer types based on five data dimensions: miRNA, methylation, single nucleotide variation, transcriptome, and copy number variation. A re-runnable parallel Linux pipeline is implemented, enabling a scalable update of the pan-cancer data at the backend.

TCGAnalyzeR provides a user-friendly web framework for integrative, large-scale analyses of genomic and clinical data of 33 cancer types from TCGA. The TCGAnalyzeR web interface allows cancer researchers to create subcohorts and/or gene sets of interest to filter through visualizations of the analyses.

## 5. Conclusions

TCGAnalyzeR provides a user-friendly web framework for integrative, large-scale analyses of the genomic and clinical data of 33 cancer types from TCGA. The TCGAnalyzeR web interface allows cancer researchers to create subcohorts and/or gene sets of interest to filter through visualizations of the analyses. For future work, we aim to integrate the subcohort targeting drug repurposing, miRNA, and methylation interfaces to TCGAnalyzeR. TCGAnalyzeR is freely available on the web at tcganalyzer.mu.edu.tr (accessed on 28 December 2023).

## Figures and Tables

**Table 1:** Comparison of TCGA visualization tools.

TCGA Visualization Tool	Login or Subscription Required for Advanced Features	Survival and Set Operation Diagrams of Selected Patient Subcohorts	Caching of Both User’s Gene and Patient Selection	Number of External TCGA Cohorts from Literature
TCGAnalyzeR http://tcganalyzer.mu.edu.tr (accessed on 28 December 2023)	No	Yes	Yes	123
cBioPortal [5,6] https://www.cbioportal.org/ (accessed on 28 December 2023)	Yes	No	Yes	0
Xena [8] https://xenabrowser.net/ (accessed on 28 December 2023)	Yes	Yes	No	0
OncoKB [9] https://www.oncokb.org/ (accessed on 28 December 2023)	Yes	No	No-only genes	0
ICGC cohort analysis [10] https://dcc.icgc.org/analysis (accessed on 28 December 2023)	No	Yes	Yes	0
Coral web application [11] https://coral.caleydoapp.org (accessed on 28 December 2023)	Yes	No	No	3

## Data Availability

The data and the codes presented in this study are available online at portal.gdc.cancer.gov (accessed on 28 December 2023), tcganalyzer.mu.edu.tr (accessed on 28 December 2023), and https://github.com/talipzengin/TCGAnalyzeR (accessed on 28 December 2023).
